# Fungal Innate Immunity Induced by Bacterial Microbe-Associated Molecular Patterns (MAMPs)

**DOI:** 10.1534/g3.116.027987

**Published:** 2016-03-29

**Authors:** Simon Ipcho, Thomas Sundelin, Gitte Erbs, H. Corby Kistler, Mari-Anne Newman, Stefan Olsson

**Affiliations:** * Department of Plant and Environmental Sciences (PLEN), University of Copenhagen, Denmark; † USDA ARS Cereal Disease Laboratory, St. Paul, Minnesota

**Keywords:** fungal–bacterial interaction, innate immunity, MAMPs, transcriptomics

## Abstract

Plants and animals detect bacterial presence through Microbe-Associated Molecular Patterns (MAMPs) which induce an innate immune response. The field of fungal–bacterial interaction at the molecular level is still in its infancy and little is known about MAMPs and their detection by fungi. Exposing *Fusarium graminearum* to bacterial MAMPs led to increased fungal membrane hyperpolarization, a putative defense response, and a range of transcriptional responses. The fungus reacted with a different transcript profile to each of the three tested MAMPs, although a core set of genes related to energy generation, transport, amino acid production, secondary metabolism, and especially iron uptake were detected for all three. Half of the genes related to iron uptake were predicted MirA type transporters that potentially take up bacterial siderophores. These quick responses can be viewed as a preparation for further interactions with beneficial or pathogenic bacteria, and constitute a fungal innate immune response with similarities to those of plants and animals.

In an environment rich in potentially pathogenic micro-organisms, the survival of higher eukaryotic organisms depends on efficient pathogen sensing and rapidly mounted defense responses. Such protective mechanisms are found in all multicellular organisms and are collectively called innate immunity. Innate immunity is the first line of defense against invading micro-organisms in vertebrates and the only line of defense in invertebrates and plants. Innate immunity in both plants and animals has been reviewed (Alexander and Rietschel 2001; Chisholm *et al.* 2006; Janeway and Medzhitov 2002; Jones and Dangl 2006; Newman *et al.* 2013) and it has become clear that although physiologically different, plants and animals share similarities, yet have distinct differences in their defense reactions against microbial pathogens (Ausubel 2005; Hunter 2005; Iriti and Faoro 2007; Nürnberger *et al.* 2004; Zipfel and Felix 2005). Both types of organism use Pattern Recognition Receptors (PRRs) to recognize various conserved structures from the bacterial cell that are commonly referred to as MAMPs, of which flagellin (FLG), lipopolysaccharides (LPS), and peptidoglycans (PGN) are well described (Newman *et al.* 2013). Furthermore, host-derived molecules, the so-called damage-associated molecular patterns (DAMPs), are often released after an infection. These can serve as signals for immunity (Newton and Dixit 2012). The PRRs contain various ligand-binding domains that perceive MAMPs or DAMPs (Zipfel 2014). Toll-like receptors (TLR), one common class of PRRs found in both animals and plants, comprise a family of transmembrane receptors that have an extracellular leucine-rich repeat (LRR) domain, by which MAMPs are recognized and the innate immune response elicited (Ausubel 2005; Zipfel and Felix 2005; Newton and Dixit 2012). The overall innate immune response consists of triggering a signaling cascade that activates reactive oxygen species (ROS), reactive nitrogen species, transcription factors, defense-related genes, and effectors (Ausubel 2005; Hunter 2005; Iriti and Faoro 2007; Nürnberger *et al.* 2004; Zipfel and Felix 2005).

Iron is an important cofactor for innate immunity, growth, defense, and virulence for many organisms (Cherayil 2011; Drakesmith and Prentice 2008; Ganz 2009; Haas 2012; Lopez-Berges *et al.* 2013). Microbes secrete high affinity siderophores called enterobactins that acquire iron (Raymond *et al.* 2003). In case of limited iron supply, some organisms produce specialized enterobactin transporters that recognize nonself siderophores and “steal” them from competing organisms (*e.g.*, MirA siderophore transporter from *Aspergillus nidulans*) (Haas *et al.* 2003). In plants, iron mediates an oxidative burst as part of the defense response (Liu *et al.* 2007) and in mammals, iron is sequestrated from invading microbes (Cherayil, 2011; Drakesmith and Prentice 2008; Ganz 2009). In the intestines, innate immune response-triggered iron sequestration plays an important role both for selecting beneficial commensals and restricting iron access to pathogens (Markel *et al.* 2007).

Previous transcriptomics studies have shown that fungi react to the presence of bacteria (Mathioni *et al.*, 2013; Mela *et al.* 2011; Schroeckh *et al.* 2009; Deveau *et al.* 2015; Gkarmiri *et al.* 2015). Despite this, very little is known about the mechanism of bacterial detection in fungi at the molecular level, and if this detection leads to an innate immune response that occurs within hours after exposure to MAMPs (Ausubel 2005; Zipfel and Felix 2005). Generally, all the previous studies employed much longer times between the start of confrontation and sampling for analysis than this study. Attempts to identify the classical PRRs containing LRR domains in fungi using bioinformatics have been unsuccessful (Soanes and Talbot 2010). Since it had previously been shown for *Candida albicans* that recognition of bacterial PGN by an intracellular LRR-containing adenylate cyclase switches the fungus from budding yeast to hyphal growth (Xu *et al.* 2008), the authors speculated that microbial recognition in fungi is potentially undertaken by the LRR-containing adenylate cyclases, which are uniquely found in fungi (Soanes and Talbot 2010). Other authors have hypothesized that fungal signal transduction ATPases with WD-repeat domains (STANDclass proteins), which have similarities to the classical PRRs, could be responsible for bacterial detection (Paoletti and Saupe 2009). Furthermore, fungal PRRs for bacterial MAMPs could be using one of the less common recognition motifs or (a) completely novel motif(s).

Fungal hyphal cells in natural environments are likewise directly exposed to both beneficial commensal bacteria and potential pathogens, and are able to react to them accordingly (Frey-Klett *et al.* 2011). We hypothesize that fungi are able to recognize bacterial MAMPs and initiate rapid transcription responses similar to those characterized in other eukaryotes. To investigate this hypothesis, we exposed *Fusarium graminearum* to the bacterial MAMPs; FLG, LOS (lipo-oligosaccharides, LPS without the O-antigen), and PGN, each having a different molecular structure. Using bacterial MAMPs has the advantage that there are no whole bacteria interfering or causing distress in the fungus, as observed in previous experiments (Mathioni *et al.* 2013; Mela *et al.* 2011; Gkarmiri *et al.* 2015; Newton and Dixit 2012). We performed a full RNA-seq transcriptomics analysis in a time course after MAMP exposure for 1, 2, and 4 hr, which should capture the most dramatic and early transcript changes, similar to both a mammalian and a plant innate immune response. We report for the first time, based on the transcriptome analysis, that a fungus has an immune system, and that it recognizes and responds quickly to bacterial MAMPs by increasing its mitochondrial activity, iron sequestration, as well as up-regulating genes encoding proteins involved in defense, secondary metabolism, and amino acid production; all known responses toward bacteria in other eukaryotes.

## Materials and Methods

### Culture of Fusarium graminearum and physiological tests

The *F. graminearum* wild-type strain PH1 (NRRL31084) (Cuomo *et al.* 2007) was maintained on Defined *Fusarium* Medium (DFM) (Yoder and Christianson 1998) (where the glucose content was reduced to 1.25% and the urea was replaced with 10 mM asparagine) at 21° in the dark, and shaking at 150 rpm if necessary.

FLG (100 ng/ml) was obtained from Invivogen (tlrl-pstfla; CA). LOS (Silipo *et al.* 2005) and PGN (Erbs *et al.* 2008) isolated from *Xanthomonas campestris* pv. *campestris* strain 8004 were used at 50 µg/ml. The MAMPs were dissolved in sterile MilliQ water. The technical sheet of FLG indicated that the product was “ultrapure” without the mention of any additives. However, the fungal transcriptomic responses to FLG showed elevated sugar metabolism. Direct inquiries with Invivogen confirmed the addition of sucrose (1 µg Flagellin/mg of sucrose). Since PGN and LOS were extracted “in-house,” we are confident of their purity and that their regulated genes were not affected by contaminants (Erbs *et al.* 2008; Silipo *et al.* 2005).

The edges of fungal cultures on agar were treated with MAMPs solution and the exposed hyphae were observed for morphological effects (detailed protocol in Supplemental Material, File S1).

The fungus membrane and mitochondrial membrane potential when elicited with MAMPs was measured with the fluorescent dye DiOC7(3) (D0929 SIGMA) (detailed protocols in File S1).

### MAMPs treatment, RNA extraction of F. graminearum, and data analysis

Fungal mycelia were produced as described in the File S1 and treated with 3 ml of each MAMPs solution or 3 ml sterile MilliQ water as the control (detailed in File S1). RNA extraction and DNase treatment were performed using the Qiagen RNeasy Plant Mini Kit and Qiagen RNase-free DNase set (Germany) using the manufacturer’s instructions.

Total RNA was submitted to the University of Minnesota Genomics Center (UMGC) for library creation using the Truseq RNA v2 kit (Illumina) and high-throughput sequencing with the HiSequation 2000 (Illumina), generating at least 8 million 50 bp pair end reads. The sequencing results were analyzed using the Tuxedo analysis suit pipeline (Trapnell *et al.* 2012) as described in File S1.

Functional Categories (FUNCAT) enrichment analysis was performed as previously described (Seong *et al.* 2008). Promoter sequences from significantly regulated genes were analyzed for potential regulating transcription factors using the MEME analysis suite (Bailey *et al.* 2009) and TOMTOM (Gupta *et al.* 2007) (more details in File S1).

The PAST analysis software (Hammer *et al.* 2001) was used for correlation studies. Principal Component Analysis (PCA) with a correlation matrix was used to compare publically available transcriptomics data with the results from this study. Pearson’s correlation was used to compare repeated RNA-seq experiments to test for reproducibility. Detailed protocols can be found in File S1.

### Data availability

The authors state that all data necessary for confirming the conclusions presented in the article are represented fully within the article. The Illumina fastq files and selected processed cuffdiff files were uploaded to NCBI GEO (GSE65311).

## Results

Fungal hyphae grown onto agar containing MAMPs showed no distinctive morphological changes at both macroscopic and microscopic levels (Figure S1).

**Figure 1:**
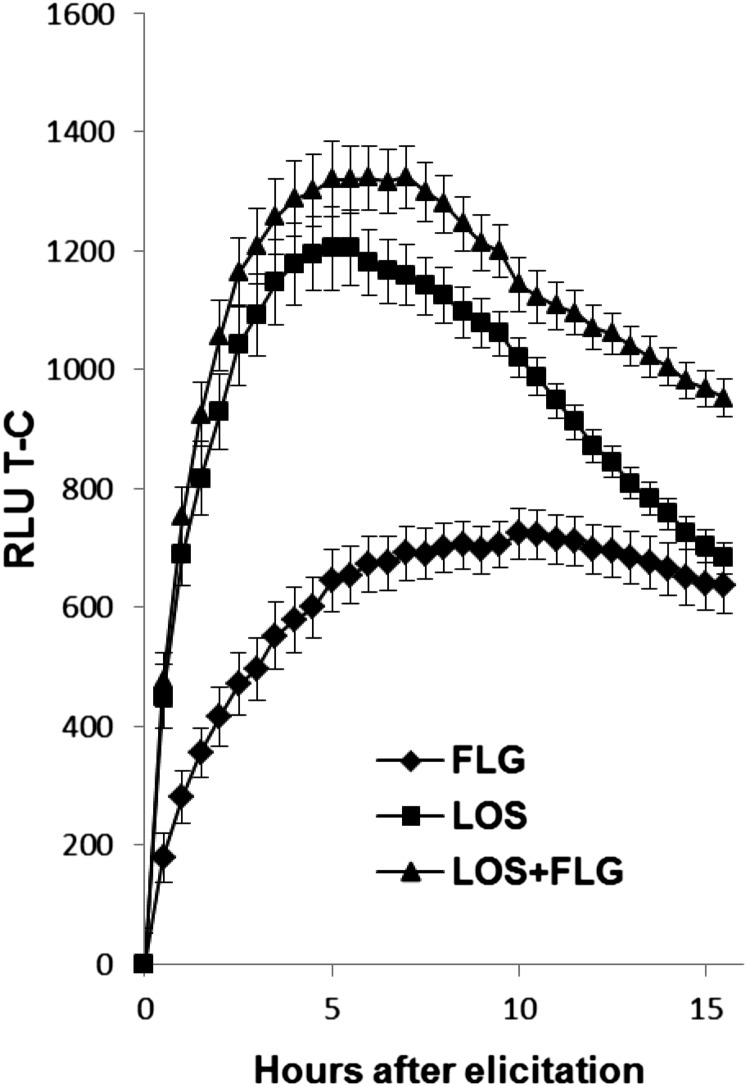
Effect of MAMPs on fungal membrane potential. A dye that incorporates into live membranes and fluoresces in response to membrane polarization was used to investigate if fungi recognize MAMPs. Upon exposure to the elicitors, the membrane polarization increased sharply during the first 2 hr. The net effect is shown as Relative Fluorescence Units (RLU) for treatment minus RLU-control (RLU T-C). N= 6; Error bars = SE. FLG, flagellin; LOS, lipo-oligosaccharides; MAMPs, microbe-associated molecular patterns.

Innate immunity reactions in animal systems generally lead to increased glycolysis, most likely in combination with a mitochondrial hyperpolarization (Widlansky *et al.* 2010; Walker *et al.* 2014). To test if similar hyperpolarization occurs in fungal mitochondria and identify the timing of fungal responses to the bacterial MAMPs exposure, DiOC7(3), a dye that stains live membranes and becomes fluorescent proportionally to the level of the membrane polarization (especially mitochondrial), was used on cultures that were treated with either FLG, LOS, or a combination of FLG and LOS. The net hyperpolarization compared to controls increased sharply during the first 2 hr and then decreased with time (Figure 1). Intriguingly, coelicitation with both FLG and LOS generated a response that was slightly higher than with LOS and FLG alone. PGN was not tested due to limited supplies.

A preliminary transcriptomics study was performed to verify if the innate immune response in *F. graminearum* follows the same timing as seen in a previous mammalian and plant studies (Amit *et al.* 2009), and as seen by the hyperpolarization response (above). The transcriptome of the fungus was sequenced after exposure to FLG for 4 hr, 10 hr, and 20 hr (data not shown). This initial transcriptomic study, as well as the membrane potential results, indicated that the response to the MAMPs was most active prior to 4 hr post elicitation and thus, the fungus was exposed for 1 hr, 2 hr, and 4 hr to FLG, LOS, or PGN for the main transcriptomics study.

**Figure 2:**
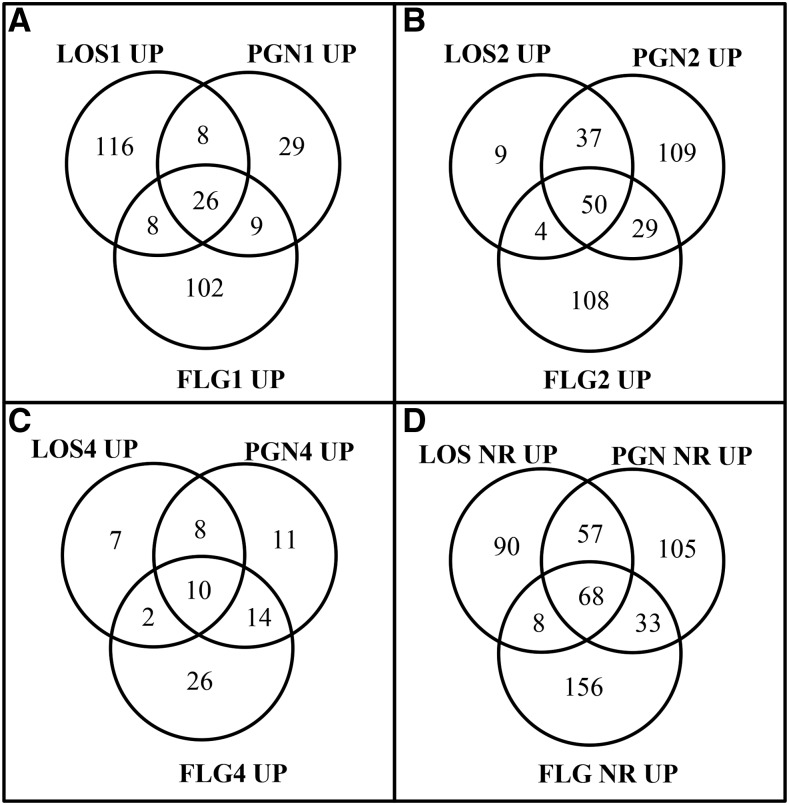
The number of genes differentially induced by MAMPs. The Venn diagram shows the number of significantly up-regulated genes identified when the fungus was treated with either LOS, PGN, or FLG, and compared against a water control. (A) Genes regulated after 1 hr. (B) Genes regulated after 2 hr. (C) Genes regulated after 4 hr. (D) The Venn diagram shows the number of nonredundant genes that was induced by each MAMP at any one of the tested times. FLG, flagellin; LOS, lipo-oligosaccharides; MAMPs, microbe-associated molecular patterns; PGN, peptidoglycans.

The significantly and differentially up-regulated genes (referred to as induced genes from here on) from the three MAMP treatments (Table S1, Table S2, and Table S3) are illustrated with a Venn diagram (Figure 2). The numbers showed that each MAMP triggers a different transcript profile. LOS rapidly induced 116 specific genes after 1 hr which were dramatically reduced at later times (Figure 2). PGN-specific genes were induced slowly and peaked to 109 genes after 2 hr (Figure 2). FLG induced numerous specific genes at both 1 hr (102 genes) and 2 hr (108 genes), but dropped to 26 genes after 4 hr. The most common genes to all three MAMPs were identified at 2 hr post inoculation with 50 genes. The list of all genes induced by LOS, PGN, and FLG at any of the tested times was compiled and redundancies removed for a global overview of the MAMP transcriptomic responses. Comparisons of the nonredundant list (Figure 2D) showed that 68 genes were commonly induced by the MAMPs. LOS and PGN shared 57 genes, while PGN and FLG shared 33 genes, and FLG and LOS had 8 genes in common.

**Figure 3:**
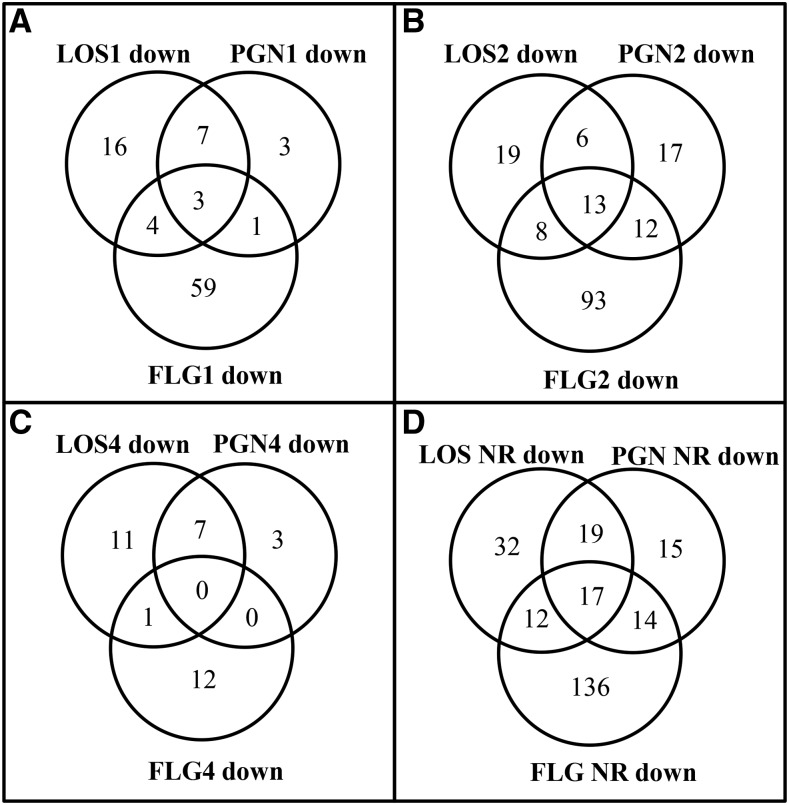
The number of genes differentially repressed by MAMPs. The Venn diagram shows the number of significantly down-regulated genes identified when the fungus was treated with either, LOS, PGN, or FLG, and compared against a water-treated culture. (A) Genes regulated after 1 hr. (B) Genes regulated after 2 hr. (C) Genes regulated after 4 hr. (D) The number of unique genes that was repressed at any of the tested times by each MAMP. FLG, flagellin; LOS, lipo-oligosaccharides; MAMPs, microbe-associated molecular patterns; PGN, peptidoglycans.

The three MAMPs also caused significant and differential down-regulation of genes that will be referred to as repressed genes from here on (Figure 3). FLG had the most repressed genes at all time-points (Figure 3). LOS and PGN-repressed genes peaked at 2 hr (Figure 3B). Commonly repressed genes were also relatively high after 2 hr of MAMPs exposure. FLG specifically repressed a total of 136 genes at any of the tested times, followed by LOS (32), and then PGN (15) (Figure 3D). LOS and PGN shared 19 repressed genes, while 17 genes were commonly repressed by all three MAMPs (Figure 3D).

The experiments with FLG and water were performed in two different laboratories and Pearson’s correlation analysis was used to compare the two results to judge the reproducibility of the datasets. The *r* value for each comparison was between 0.926–0.953 (Figure S2). The lowest correlation values were with samples from the 2 hr treatments, as could be expected, since the biological variability is highest at that time-point.

The presence of sucrose (originally unknown to us) in the commercially obtained FLG induced genes associated with sugar metabolism, and more particularly sucrose metabolism (for details see *Materials and Methods* and Table S4). Thus, the FLG transcriptomics data were clouded by sucrose-regulated genes. Therefore, the 156 and 136 genes specifically regulated by FLG will not be discussed in detail (Figure 2D and Figure 3D).

Induced and repressed genes were analyzed for functional enrichment (Table S5, Table S6, and Table S7). To simplify the analysis, only nonredundant genes will be discussed. Functional analysis of the induced common MAMP genes (Table 1 and Table S8) revealed four distinct themes: 1) transport of compounds such as transport ATPases, nonvesicular cellular import, siderophore-iron transport, and homeostasis of metal ions; 2) defensive processes such as detoxification, ABC transporters, and drug/toxin transport; 3) metabolism of hydrophobic amino acids derived from pyruvate such as valine, isoleucine, and leucine; and 4) increased mitochondrial activity (tricarboxylic-acid pathway, TCA). Conversely, only three categories [Biosynthesis of vitamins, cofactors, and prosthetic groups, tetraterpene (carotenoids) metabolism, and metabolism of sulfuric acid and L-cysteine derivatives] were significantly overrepresented among the repressed genes post enrichment analysis (Table 1 and Table S9).

**Table 1:** Functional categories that were significantly enriched (*P* < 0.01) when MAMPs-regulated genes were analyzed

Functional Category	Differentially Expressed Genes Identified	FUNCAT Related Genes in Genome	*P*-Value
Genes up-regulated			
01 Metabolism	29	3402	6.08E-04
01.01.11.02 Metabolism of isoleucine	2	25	6.46E-03
01.01.11.03 Metabolism of valine	2	21	4.58E-03
01.01.11.04 Metabolism of leucine	2	31	9.83E-03
01.07 Metabolism of vitamins, cofactors, and prosthetic groups	6	346	6.51E-03
02.10 Tricarboxylic-acid pathway (citrate cycle, Krebs cycle, TCA cycle)	3	54	2.26E-03
20.01 Transported compounds (substrates)	16	1597	3.38E-03
20.01.01 Ion transport	8	321	1.53E-04
20.01.01.01 Cation transport (H^+^, Na^+^, K^+^, Ca^2+^, NH_4_^+^, *etc*.)	8	269	4.47E-05
20.01.01.01.01 Heavy metal ion transport (Cu^+^, Fe^3+^, *etc*.)	8	99	2.39E-08
20.01.01.01.01.01 Siderophore-iron transport	7	41	8.98E-10
20.01.27 Drug/toxin transport	8	171	1.63E-06
20.03.22 Transport ATPases	4	123	2.97E-03
20.03.25 ABC transporters	4	106	1.73E-03
20.09 Transport routes	13	1237	5.82E-03
20.09.18 Cellular import	11	519	3.58E-05
20.09.18.07 Nonvesicular cellular import	8	278	5.64E-05
32.07 Detoxification	9	520	8.61E-04
34 Interaction with the environment	13	832	1.51E-04
34.01 Homeostasis	8	336	2.09E-04
34.01.01 Homeostasis of cations	8	290	7.59E-05
34.01.01.01 Homeostasis of metal ions (Na, K, Ca, *etc*.)	8	208	6.99E-06
40.10.02.02 Apoptotic program	2	27	7.51E-03
40.10.02.02.01 Apoptotic mitochondrial changes	2	10	1.02E-03
Genes down-regulated			
01.06.06.13 Tetraterpenes (carotinoids) metabolism	1	3	3.68E-03
01.07 Metabolism of vitamins, cofactors, and prosthetic groups	4	346	7.09E-04
01.07.01 Biosynthesis of vitamins, cofactors, and prosthetic groups	4	179	5.67E-05
01.20.21 Metabolism of sulfuric acid and L-cysteine derivatives	1	8	9.80E-03

The figures are representative of the 68 genes that were significant induced, and 17 genes that were significantly repressed, by all three MAMPs at any of the three studied times. MAMPs, microbe-associated molecular patterns; FUNCAT, functional category; TCA, tricarboxylic-acid.

LOS and PGN shared 57 induced genes that were not significantly regulated by FLG (Table 2). FUNCAT analysis of these 57 genes strengthened the observations in Table 1. Thus, similar themes were observed whereby the metabolism of amino acids, defense-related proteins, and energy-related genes were over represented. Additionally, increased carbon-based metabolism (C-compound and carbohydrate metabolism) and protein synthesis (rRNA processing and ribosome biogenesis) were observed (Table 2). Looking at the genes listed under the categories of Energy and C-compound and carbohydrate metabolism, it is apparent that most of them are related to the TCA and glyoxylate cycle (*e.g.*, FGSG_00176 and FGSG_09896 are probable isocitrate lyases; FGSG_07953 is a probable aconitase; FGSG_00330 is an acetyl-CoA synthase; and FGSG_08700 is a probable malate synthase).

**Table 2:** Common genes that were significantly induced (57) or repressed (19) by both LOS and PGN at any of the tested times were subject to functional category enrichment analysis

Functional Category	Differentially Expressed Genes Identified	FUNCAT Related Genes in Genome	*P*-Value
Genes up-regulated			
01 Metabolism	25	3402	1.12E-03
01.01 Amino acid metabolism	12	649	1.05E-05
01.01.03.02 Metabolism of glutamate	3	63	2.21E-03
01.01.03.02.01 Biosynthesis of glutamate	3	41	6.34E-04
01.01.06.04 Metabolism of threonine	2	18	2.45E-03
01.01.06.04.02 Degradation of threonine	2	12	1.07E-03
01.01.11 Metabolism of the pyruvate family (alanine, isoleucine, leucine, and valine) and D-alanine	5	65	6.81E-06
01.01.11.02 Metabolism of isoleucine	2	25	4.71E-03
01.01.11.02.02 Degradation of isoleucine	2	10	7.36E-04
01.01.11.03 Metabolism of valine	2	21	3.33E-03
01.01.11.03.02 Degradation of valine	2	11	8.97E-04
01.01.11.04 Metabolism of leucine	5	31	1.56E-07
01.01.11.04.01 Biosynthesis of leucine	2	21	3.33E-03
01.01.11.04.02 Degradation of leucine	5	19	1.11E-08
01.02.02 Nitrogen metabolism	3	63	2.21E-03
01.02.02.09 Catabolism of nitrogenous compounds	2	28	5.89E-03
01.05 C-compound and carbohydrate metabolism	15	1547	1.17E-03
01.05.02.04 Sugar, glucoside, polyol, and carboxylate anabolism	3	85	5.17E-03
01.05.06 C-2 compound and organic acid metabolism	5	45	1.07E-06
01.05.06.07 C-2 compound and organic acid catabolism	5	39	5.15E-07
01.20.07 Metabolism of propionic acid derivatives	1	1	4.12E-03
02 Energy	11	609	3.22E-05
02.10 Tricarboxylic-acid pathway (citrate cycle, Krebs cycle, and TCA cycle)	4	54	7.04E-05
02.16 Fermentation	5	109	8.39E-05
02.16.11 Propionate fermentation	3	5	6.61E-07
11.04 RNA processing	7	442	2.16E-03
11.04.01 rRNA processing	6	187	1.16E-04
12 Protein synthesis	7	439	2.08E-03
12.01 Ribosome biogenesis	7	255	8.05E-05
32.05.03 Defense-related proteins	3	92	6.44E-03
40.10.02.01 Antiapoptosis	2	31	7.19E-03
Genes down-regulated			
02 Energy	5	609	1.13E-03
02.16 Fermentation	2	109	9.65E-03
20.01.03 C-compound and carbohydrate transport	3	332	9.99E-03
20.01.11 Amine/polyamine transport	2	58	2.82E-03
20.03 Transport facilities	5	808	3.94E-03
20.03.02 Carrier (electrochemical potential-driven transport)	3	142	9.11E-04
20.03.02.03 Antiporter	2	75	4.68E-03
20.03.02.03.01 Proton driven antiporter	2	45	1.71E-03

The results show those categories that were significantly enriched (*P* < 0.01). LOS, lipo-oligosaccharides; PGN, peptidoglycans; FUNCAT, functional category; TCA, tricarboxylic-acid; rRNA, ribosomal RNA.

Conversely, LOS and PGN also shared 19 repressed genes that showed functional enrichment for transport (C-compound and carbohydrate transport, amine/polyamine transport, and proton driven antiporter) and fermentation.

LOS induced 90 specific genes and PGN induced 105 specific genes (Figure 2D). FUNCAT analysis (Table S10) was used to compare the molecular events specific to each of the mentioned MAMPs. Among the major differences, LOS selectively induced more genes related to the metabolism of methionine and cysteine. As a result, more genes related to sulfur metabolism, homeostasis of anions, and transport of anions were observed to be selectively induced. On the other hand, PGN selectively induced the metabolism of proline and the ringed amino acids phenylalanine, tyrosine, and tryptophan.

PGN induced more genes that respond to external stimuli (Table S10) as compared to LOS. Two of these genes (FGSG_05006 , a G protein-coupled receptor and FGSG_01298 , a transcriptional repressor) would have major downstream effects on gene regulation and could potentially explain the observation of more significantly regulated genes within the categories of transcription, protein synthesis, protein fate, protein with binding function or cofactor requirement, and metabolism of vitamins, cofactors, and prosthetic groups. Additionally, PGN significantly induced three MFS (major facilitator superfamily) proteins at higher level than the other MAMPs (FGSG_03725 , FGSG_07564 , and FGSG_08823 , which are classified under drug/toxin transport and detoxification by export).

All the MAMPs triggered increased energetic demand as mentioned above. However, LOS specifically induced an additional alcohol dehydrogenase 1 (FGSG_02034 ) that peaks at 1 hr as compared to the other treatments (Table S10; 02.16 fermentation).

Since iron sequestration is a key element for both animal (Cherayil 2011; Drakesmith and Prentice 2008; Ganz 2009; Markel *et al.* 2007) and plant (Liu *et al.* 2007) innate immunity responses, genes involved in iron metabolism were investigated. These genes showed more prominent expression levels in PGN and FLG as compared to LOS (Table S11). The description of the induced genes showed that approximately half of them were described as MirA siderophore transport genes. Another five genes within the list were described as ferric reductases, which are required for iron assimilatory pathways (Saikia *et al.* 2014). Additionally, three genes encoding enzymes for the production of fungal siderophores (FGSG_03747 , FGSG_04333 , and FGSG_05371 ) were significantly induced when exposed to MAMP(s). Further examination of selected genes related to siderophore biosynthesis confirmed that iron metabolism is quickly up-regulated, as early as 1 hr post elicitation with MAMPs (Table S12) (Yasmin *et al.* 2012).

Table S13 illustrates a selection of 15 genes related to secondary metabolism and defense, and induced by at least one of the MAMPs. Half of them encode multidrug resistance proteins or ABC transporters that usually provide the ability to export endogenous or exogenous toxins (Coleman and Mylonakis 2009). A polyketide synthase and two monooxygenases, which often indicate the production of secondary metabolites, were found to be induced. Other genes of interest were a PR-1 class of pathogen-related proteins, which respond to bacterial pathogens in plants (Van Loon and Van Strien 1999), and finally a gene related to aliphatic nitrilase, which is often involved in detoxification of nitriles to their respective carboxylic acids and ammonia (O’Reilly and Turner 2003).

**Figure 4:**
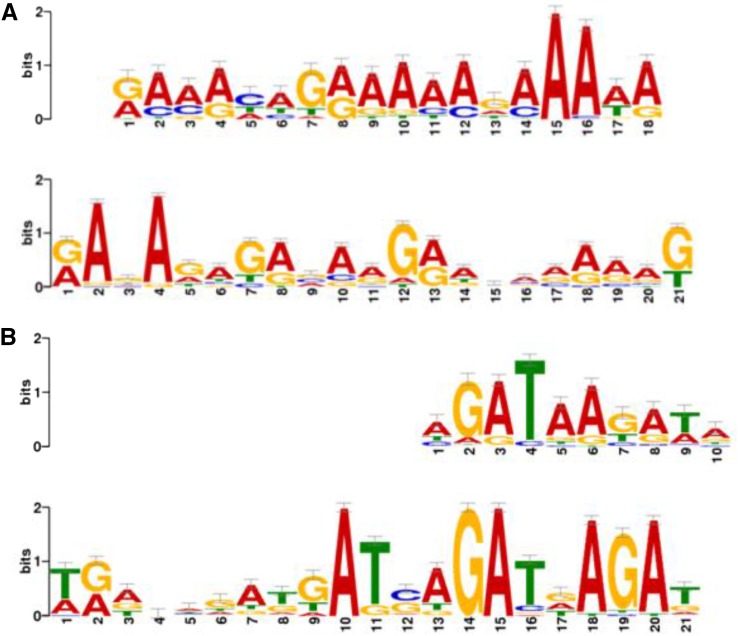
Potential transcriptional factor binding sites on the promoter region of MAMP-induced genes. The promoter region of the 68 common MAMP-induced genes and the 57 genes induced by both PGN and LOS were searched for transcription factor binding motifs to potentially identify the transcription factors regulating them. Two major element binding motifs have been identified. (A) All of the common MAMP genes and 16 of the genes induced by both PGN and LOS had a binding motif that potentially binds to the transcription factor AZF1 in yeast, with FGSG_16816 (formerly FGSG_13123) as an ortholog in *F. graminearum*. (B) 26 genes of the common MAMP genes had promoter sequence similarities to a GATA binding motif that matched the promoter sequence for GLN3 in yeast. The *F. graminearum* transcription factors FGSG_09565 , FGSG_08634 , and FGSG_16452 (formerly FGSG_05073) have similarities to GLN3. LOS, lipo-oligosaccharides; MAMPs, microbe-associated molecular patterns; PGN, peptidoglycans.

The promoter region of the common MAMPs genes and 57 genes induced by both LOS and PGN were analyzed by the MEME analysis suite to identify their potential regulators. All the 68 common MAMPs genes had promoter binding motifs that matched the binding motif of the AZF1 transcription factor (*P* = 0.00017) in yeast (Figure 4A). A sequence similarity search of AZF1 in *F. graminearum* showed that the fungal genome contained a protein described as a finger protein related to AZF1 (FGSG_16816 ; formerly FGSG_13123) with 48% similarity (Table S14).

Out of the 68 common MAMP genes, 26 genes had a GATA binding motif in their promoter region (Figure 4B) that matched the yeast GLN3 transcription factor binding site (*P* = 0.00058). Seven of these 26 genes were genes related to iron metabolism as listed in Table S11. A sequence similarity search of GLN3 protein against the *F. graminearum* genome sequence indicated that these 26 genes could be regulated by FGSG_09565 , a probable siderophore regulator (57% identity); FGSG_16452 (formerly FGSG_05073), an ASD4 related protein (60% identity); and/or FGSG_08634 (FgAreA) a global nitrogen regulator (77% identity) (Table S14). Of the 57 genes commonly induced by both LOS and PGN, 16 genes had promoter sequences that had a transcription binding site that matched the AZF1 transcription factor (*P* = 0.00001), as described above.

**Figure 5:**
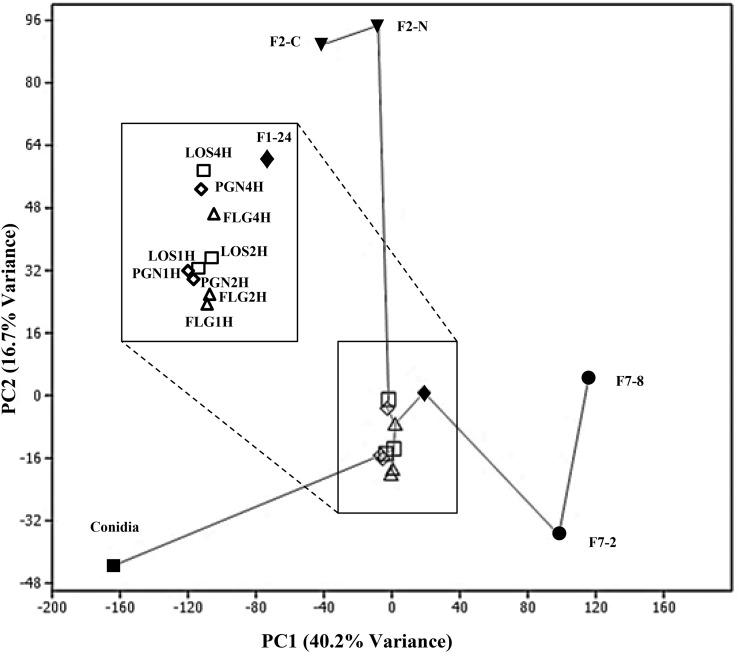
Principal component analysis comparison of published *F. graminearum* transcriptome studies with MAMP transcriptome profiles. The gene expression fold change of treated *F. graminarum* compared to control from the published *F. graminearum* transcriptomics studies of Carbon/Nitrogen starvation (F2-C/N), nongerminated conidia (Conidia), 24 hr post plant infection (F1-24), and germinated conidia at 2 or 8 hr (F7-2/8) was used to compare the gene expression fold change compared to control profiles of the MAMPs study. The broken lines connect to an enlarged picture of the area where the MAMPs-treated fungal data and fungal–plant infection data have clustered closely. MAMP datasets are named according to the treatment and exposure time (*e.g.*, FLG1H is data of the changed transcriptome of a fungus exposed to FLG for 1 hr as compared to control). FLG, flagellin; LOS, lipo-oligosaccharides; MAMPs, microbe-associated molecular patterns; PC, principal component; PGN, peptidoglycans.

To better understand how the MAMP global transcriptomic responses are different to other studied transcriptomic responses of *F. graminearum*, a PCA (Abdi and Williams 2010) with correlation matrix was used to compare the MAMPs datasets against other publicly available transcriptomic experiments (see Figure 5 and File S1 for interactions and comparisons of the transcriptomics studies). These expression datasets were chosen as the fungus in these experiments was physiologically close to our experimental settings (same strain and short time treatments). A PCA compares the various datasets, captures the elements contributing to the most variations in the data as components, and visually displays how these components are spread out among the samples. As illustrated in Figure 5, Principal Component 1 (PC1) captured 40.2% variance and PC2 captured 16.7% variance. PC1 resolved the samples mainly according to their change in physiological state. The changes in physiological state compared was mycelium to nongerminated conidia (Conidia), our data for untreated mycelium to MAMPs-treated mycelium (MAMPs), vegetative mycelium in culture to parasitic mycelium *in planta* (F1-24), control mycelium on complete media to carbon/energy starved mycelium (F-C), control mycelium on complete media to nitrogen starved mycelium (F-N), and ungerminated conidia to germinated conidia (F7). PC2 resolved the carbon (C) and nitrogen (N) starved samples from the other treatments. The gray lines represent the minimum spanning tree linking the treatments with the most similar transcriptomics profile. For clarity, the closely clustered MAMP samples and fungal plant infection transcriptomic study have been enlarged (Figure 5).

Interestingly, the plant infection transcriptomics profile (F1-24) clustered very closely to the MAMPs treatments. PC3 and PC4 captured 14.1% and 9.3% variance, respectively (Figure S3). The plant infection sample was distanced from the MAMPs cluster with approximately 7% and 3.5% variance along PC3 and PC4, respectively. The starvation (C/N) samples were brought nearer to the MAMPs cluster along PC3 and PC4, whereas the conidia and germination transcriptomes were further distanced. The MAMPs samples showed slight changes but still clustered very close to each other, showing that there is very little difference between their overall transcriptome profiles across these four PCs.

## Discussion

*F. graminearum* was exposed to MAMPs to investigate if a fungus can express innate immune molecular cues similar to those demonstrated in plants and animals. As observed, MAMPs do not cause observable morphological effects (Figure S1) but affect the fungus at subcellular levels. Using a fluorescent dye, membrane polarization reflecting mitochondrial activity was found to rapidly increase upon inoculation with MAMPs in line with what is detected in animal cells (Widlansky *et al.* 2010). When the fungus was challenged with both FLG and LOS, the response was slightly higher than the individual response to LOS, and showed that the individual LOS and FLG responses were neither additive nor synergistic.

An increased mitochondrial activity is often connected with innate immunity in animals (Arnoult *et al.* 2011; Walker *et al.* 2014), and conservation of this response was further confirmed by this fungal transcriptomic study. All the MAMPs triggered increased energetic demands with the activation of the mitochondrial TCA cycle, glyoxylate cycle, fermentation, and a high level of ATPase transporter expression. Increased energetic demand through fatty acid degradation has also been shown when the plant pathogenic fungus *Rhizoctonia solani* was under bacterial stress (Gkarmiri *et al.* 2015). Additionally, elevated transcription of ATP/ADP porter (FGSG_06021 ) also supports the idea of increased energetics demand. LOS additionally induced an alcohol dehydrogenase 1 (FGSG_02034 ) that peaked at 1 hr, yet transcripts of this gene only accumulated to very low levels in response to PGN. This also potentially explains the earlier increase in gene induction after LOS treatment (1 hr) compared to induction after treatment with PGN (2 hr) (Figure 2). The assumed higher energy demand is also reflected by the membrane polarization assay (Figure 1). The quick rise in FLG-induced genes at 1 hr could partly be explained by the presence of sucrose in the product. These fast responses (within 2 hr) are general for innate immune responses in animal and plant systems (Amit *et al.* 2009; Newman *et al.* 2013). The pattern of different gene expression responses to different MAMPs and a core set of responding genes found by us are similar to those recently described in transcriptomic studies on animal innate immunity (Amit *et al.*, 2009; Yang *et al.* 2015).

Despite having used three different MAMPs, each with different molecular configurations (even with sugar in the FLG), it is interesting to observe that the PCA results showed that changes in the transcriptome due to the MAMP treatments did not diverge much across the first four PCs (80.3% variance) when compared to the other transcriptome changes (Figure 5 and Figure S3). This indicates that very similar groups of genes are regulated by exposure to any of the three MAMPs.

Furthermore, proximity of the fungal–plant infection transcriptome profile and the MAMP treatments profile along PC1 and PC2 (56.9% variance) (Figure 5) suggest that there is an overlap of genes with similar expression profiles that are involved in both fungal growth *in planta* and fungal growth in the presence of bacteria. The similarities between this study to plant infection studies, in functional groups of genes up-regulated, include the production of efflux pumps (Morrissey and Osbourn 1999), up-regulation of secondary metabolism genes (Keller *et al.* 2005), and uptake of nutrients such as iron (Greenshields *et al.* 2007), and indicate that the same mechanisms for fungal pathogenicity on plants may be used for fungal defense against bacteria.

The production of efflux pumps suggests that *F. graminearum* could be mounting a defensive response in anticipation of antifungal compounds that may be introduced in the immediate environment by bacteria. Alternatively, the fungus could be secreting antimicrobial compounds which will also require transporters and energy. Many *Fusarium* species are known to produce antimicrobial compounds such as fusaric acid and aurofusarin (Duffy *et al.* 2003; Trail 2009). Either scenario indicates a defensive reaction in preparation for a bacterial confrontation. Increased production of the efflux pumps is further supported by the FUNCAT analysis, which showed increased metabolism of mostly hydrophobic amino acids that can be linked to the increased production of transmembrane proteins and is required for the synthesis of various types of transporters (de Planque and Killian 2003).

The polyketide synthase PKS11 is believed to create a partially or fully reduced polyketide with methylation that can potentially generate secondary metabolites (Hansen *et al.* 2012). Interactions of *A. nidulans* with soil bacteria have shown the induction of expression of gene clusters that contained PKS responsible for the production of secondary metabolites (Schroeckh *et al.* 2009). Direct contact of bacteria with the hyphae of *Magnaporthe oryzae* also stimulated secondary metabolism through increased expression of cytochrome P450 (Mathioni *et al.* 2013). It is also to be noted that the fungus did not react to MAMPs with aggressive up-regulation of known secondary metabolism gene clusters, suggesting that either the fungus produces antimicrobial compounds that are still unknown or that the initial detection of MAMPs triggers a defensive mechanism that awaits further signals from the bacteria to respond accordingly.

Previous fungal–bacterial studies have also shown that bacterial presence (Mathioni *et al.* 2013) or the presence of bacterial metabolites (Mela *et al.* 2011) can repress the expression of cell wall degrading enzymes such as xylosidases, alpha-glucosidase sorbitol dehydrogenases, and endoglucanase. Nevertheless, in the presence of a nonpathogenic bacterium, cell wall degrading enzymes were expressed (Mathioni *et al.* 2013). Similarly, in this study, since the fungus was not inhibited by live bacteria, we observed the significant differential up-regulation of transcripts for enzymes typically associated with cell wall degradation, such as FGSG_11326 , a probable glucan 1,4-alpha-glucosidase; FGSG_03462 , a probable alpha-glucosidase; FGSG_03410 , related to beta-glucosidase; and FGSG_05374 , related to cellobiose dehydrogenase. We suspect that these enzymes catalyze the cleavage of the glycosidic beta 1-4 bonds between N-acetylglucosamine and N-acetylmuramic acid sugars within the peptidoglycan cell walls (Newman *et al.* 2013), and hence speculate that pathogenic bacteria have developed effectors that repress such enzymes to protect themselves.

Upon exposure to MAMPs, the fungus induced iron metabolism and even upregulated transcripts for transporters for the uptake of bacterial siderophores (Table S11). This is not surprising given the critical involvement of iron to various cellular processes and virulence (Dixon and Stockwell 2013; Drakesmith and Prentice 2008; Haas 2012). Iron acquisition has also been shown to be critical for the successful establishment in the rhizosphere and bacteria, capable of fierce competition for iron, typically limit fungal growth, as has been shown for many PGPRs (plant growth promoting rhizobacteria) (Beneduzi *et al.* 2012). *Ustilago maydis* is also known to compete for iron when in the presence of other fungi (Jonkers *et al.* 2012). In plants, iron is responsible for mediating ROS, which is crucial to the plant’s innate immune response (Liu *et al.* 2007), and in mammals, iron is sequestered away from bacterial reach as part of the innate immune response during bacterial infection (Ganz 2009; Johnson and Wessling-Resnick 2012; Ong *et al.* 2006). Thus, monopolizing this cofactor in the presence of a competitor has two main implications: 1) the competing organism (bacteria) will be disadvantaged metabolically with less iron, and 2) the fungus can have a competitive advantage for bacterial warfare. It may also serve as a mechanism for selecting beneficial commensal bacteria as these can be expected to tolerate the competition. Innate immune response-triggered iron sequestration also plays a role both in selecting beneficial commensals and restricting iron access for pathogens in the intestines (Markel *et al.* 2007). This further demonstrates the ability of fungi to recognize other organisms and prepare themselves for defense in manners that are similar to the mammalian innate immune system.

Promoter sequence analysis showed that 84 genes were potentially regulated by the yeast AZF1 transcription factor. Similar results have also been uncovered with the transcriptomics analysis of the interaction between the nonpathogenic strain of *Lysobacter enzymogenes* and *M. oryzae* (Mathioni *et al.* 2013). AZF1 is known to activate genes related to cell wall maintenance (Newcomb *et al.* 2002), and bacteria are known to secrete chitinases that soften the fungal cell wall, leading to infection (Moebius *et al.* 2014). Additionally, the noncontact study between *A. niger* and *Collimonas fungivorans* also showed signs of attacks on the fungal cell wall (Mela *et al.* 2011). Since the cell wall is the first line of defense from pathogens, we speculate that, upon detecting bacteria, fungi modify their cell wall as a defensive mechanism. Therefore, it may be advantageous for bacteria such as *L. enzymogenes* to inhibit fungal cell wall repair genes.

Among the 68 common MAMP-induced genes, 26 genes (12 annotated) were also found to have GATA transcription factor binding sites that could be regulated by the yeast transcription factor GLN3. GLN3 is known to be an activator of nitrogen metabolism genes during conditions of low nitrogen supply (Minehart and Magasanik 1991) and when treated with the antifungal compound rapamycin (Feller *et al.* 2013). Thus, the probable nitrogen catabolic enzyme regulatory protein FgAreA (FGSG_08634 ) could be the best candidate as a regulator of these 26 genes (Giese *et al.* 2013). There is also precedence that showed that when fungi detect the presence of bacterial metabolites, both organisms scavenge for nitrogen compounds (Mela *et al.* 2011). However, out of the 12 genes with described functions, only the putative amino acid permease (FGSG_04943 ) is relevant to nitrogen metabolism. Therefore, the two previously described GATA binding transcription factors could be potential regulators, especially considering that genes related to iron metabolism are also regulated by a GATA transcription factor (Table S14 and Table S15).

A total of 762 genes were regulated by the tested MAMPs (Figure 2D and Figure 3D); during early nonpathogenic bacterial attachment to *M. oryzae*, when the fungus is likely exposed to a variety of MAMPs, 765 genes were regulated (Mathioni *et al.* 2013). Despite the fact that the FLG treatment was contaminated with sucrose, which positively influenced the number of regulated genes in this study, and the fact that the *M. oryzae* study used slightly different significance settings, the number of regulated genes between these two studies correlate. Additionally, similar biological processes were also identified as mentioned before.

As previously mentioned, innate immunity of plants and animals shares striking similarities. The transcriptomic results were data-mined for additional cues of innate immunity. PGN and LOS (Table S1 and Table S2) induced thioredoxin encoding genes (FGSG_03180 , FGSG_03946 , and FGSG_07536 ) used in ROS defense, and suggest the production of ROS similar to in the immune systems of plants and animals (Powis and Montfort 2001). Alternatively, the increase in thioredoxin could either be a preparation for membrane leakage-induced ROS stress, commonly caused by bacterial interactions (Crowe and Olsson 2001), or just a consequence of the need to remove ROS produced by the hyperpolarized mitochondria, as often seen in inflammatory responses (Perl *et al.* 2004; Zhang *et al.* 2007). Efforts to detect ROS by exposing *F. graminearum* to MAMPs with luminol and lucigenin were unsuccessful, pointing toward these alternatives (data not shown). Similarly, antioxidants were also found to be produced when the fungus *R. solani* was exposed to bacterial antagonists (Gkarmiri *et al.* 2015)

It was also found that the putative STAND proteins (FGSG_16138 , formerly FGSG_03153; FGSG_10569 ; and FGSG_14016 ) were induced by the MAMPs. These intracellular STAND proteins have been hypothesized to be potential PRRs to detect MAMPs, and will be good candidates for functional studies of MAMPs sensing (Paoletti and Saupe 2009). Searching the databases, we could also find five genes that are predicted to encode LRR-containing adenylate cyclases (ATP pyrophosphate-lyase) or LRR-containing lyases uniquely found in fungi, and that have been speculated to be involved in recognition (Soanes and Talbot 2010) (Table S16). Of these, only FGSG_01522 was significantly induced by the MAMPs treatments (FLG H1, Table S3). Even though the identified LRR-containing adenylate cyclase transcription responses were weak, as might be expected for receptor-type proteins, they could be part of a recognition machinery if MAMPs are taken up and internalized in fungal cells as they are in animal cells (Letran *et al.* 2011; Zanoni *et al.* 2011). Interestingly, subcellular localization of MAMP receptors in intestinal epithelial cells has been suggested to facilitate the discrimination of commensal *vs.* pathogenic bacteria (Artis 2008). Since this discriminatory function can be expected to be essential for a fungal innate immunity, an internal localization of MAMP receptors might be expected. As previously mentioned, the classical LRR PRRs, which are often associated with the detection of MAMPs, have not been identified in fungi (Soanes and Talbot 2010). Our study provides additional clues toward the hunt for MAMP receptors in fungi.

### Concluding remarks

Using MAMPs allowed for a simple modeling system to study the fungal–bacterial interaction. Previous publications showed that bacterial pathogens secrete secondary metabolites that negatively affect the fungus (Mela *et al.* 2011), as well as potential effectors that modulate gene expression within the fungus (Mathioni *et al.* 2013). MAMPs are able to trigger transcriptomic responses in the fungus while showing no obvious changes in fungal growth and morphology. The optimal transcriptomic changes were observed within the first 2 hr of MAMPs exposure, similar to those seen in mammals and plants (Amit *et al.* 2009). The most characteristic responses were an induction of genes involved in iron acquisition, oxidative stress, detoxification, and secondary metabolites. All these responses can be viewed as a preparation for beneficial or pathogenic bacterial interactions, and to constitute a fungal type of innate immunity response with similarities to plant and especially animal innate immunity. Knowledge of the MAMPs responses in fungi allows for a more targeted approach in future studies, which will aim to identify the receptors of both LOS and PGN and the following signaling pathways leading to fungal immune responses.

## Supplementary Material

Supplemental Material
