# Supplementary material for: Fungal Innate Immunity Induced by Bacterial Microbe-Associated Molecular Patterns (MAMPs)
Source: G3 (Bethesda). 2016 Mar 29;6(6):1585–95. doi: 10.1534/g3.116.027987 (PMC4889655; doi:10.1534/g3.116.027987)
Supplement: Supplemental Material [file supp_6_6_1585__index.html]

Supplemental Material 

# Fungal Innate Immunity Induced by Bacterial Microbe-Associated Molecular Patterns (MAMPs)

Supplemental Material for Ipcho *et al.*, 2016

Supplemental Material

**Files in this Data Supplement:**

- Table S1 - LOS genes that are significantly regulated over 1,2 and 4 hours. (.xlsx, 96 KB)
- Table S2 - PGN genes that are significantly regulated over 1,2 and 4 hours. (.xlsx, 100 KB)
- Table S3 - FLG genes that are significantly regulated over 1,2 and 4 hours. (.xlsx, 132 KB)
- Table S4 - Sugar-related induced genes with flagellin treatment. (.xlsx, 12 KB)
- Table S5 - FUNCAT of regulated genes from LOS-MQ. (.xlsx, 42 KB)
- Table S6 - FUNCAT of regulated genes from PGN-MQ. (.xlsx, 44 KB)
- Table S7 - FUNCAT of regulated genes from FLG-MQ. (.xlsx, 49 KB)
- Table S8 - FUNCAT of 68 non-redundant common genes induced by MAMPs. (.xlsx, 19 KB)
- Table S9 - FUNCAT of 17 non-redundant common genes repressed by MAMPs. (.xlsx, 19 KB)
- Table S10 - FUNCAT of genes only expressed by LOS or PGN. (.xlsx, 21 KB)
- Table S11 - Expression of significantly induced genes related to iron transport. (.xlsx, 15 KB)
- Table S12 - Expression of selected gene related to siderophore biosynthesis. (.xlsx, 14 KB)
- Table S13 - Secondary metabolites related genes. (.xlsx, 16 KB)
- Table S14 - List of potential transcription factors regulating MAMPs genes. (.doc, 34 KB)
- Table S15 - Expression of putative MAMPs regulatory genes as identified from yeast databases. (.xlsx, 12 KB)
- Table S16 - The table below (and illustrated as a figure) shows the Log 2 fold change of genes encoding LRR-containing adenylate cyclases (ATP pyrophosphate-lyase) or lyase domains in responses to MAMPs in relation water control in *Fusarium graminearum*. (.doc, 60 KB)
- Figure S1 - The effect of MAMPs on fungal cultures was tested.MAMPs (20ul) were deposited at the edge of fungal cultures on agar plates and allowed to grow onto the MAMPs inoculated area overnight. (.pdf, 93 KB)
- Figure S2 - To validate the reproducibility of the fungal reactions, RNA-Seq expriments with flagellin (FLG) and water (MQ) were performed in two different laboratories with biological triplicates. (.pdf, 329 KB)
- Figure S3 - The figure above illustrates the relationships of the transcriptomics profiles from different experiments in relation to each other within the 3rd and 4th principle component capturing 14.1% variance and 9.3% variance respectively. (.pdf, 41 KB)
- File S1 - Supplementary materials and methods. (.pdf, 359 KB)
