# Supplementary material for: Fungal Innate Immunity Induced by Bacterial Microbe-Associated Molecular Patterns (MAMPs)
Source: G3 (Bethesda). 2016 Mar 29;6(6):1585–95. doi: 10.1534/g3.116.027987 (PMC4889655; doi:10.1534/g3.116.027987)
Supplement: Supplemental Material [file supp_g3.116.027987_TableS14.docx]

Table S14: The list of trancription factors that potentially are regulating the expression of the MAMPs genes and have motif binding sites as illustrated in Figure 4. The phenotype of each of the gene listed is highlighted if the deletion mutant of these genes ([Son et al, 2011](#_ENREF_9)) have reduced: radial growth on PDA^1^; growth on minimal media^2^; toxin production^3^; virulence on wheat heads^4^; could not be transformed^5^ and no noticeable alteration^6^.

| Gene id | Putative gene function | Related ortholog in other fungi | Function |
| --- | --- | --- | --- |
|  |  |  |  |
| FGSG_05073 ^5^ | related to ASD-4 GATA type zinc finger protein ASD4 | ASD4 | Regulates sexual development in Neurospora ([Feng et al, 2000](#_ENREF_1)) |
| FGSG_06944 ^124^ | related to HCM1 - transcription factor | HCM1 | Regulates cellular replication and life span in yeast ([Maoz et al, 2014](#_ENREF_4); [Pramila et al, 2006](#_ENREF_6)) |
| FGSG_08634 ^23^ | Nitrogen catabolic enzyme regulatory protein AreaA | AreA | Regulates nitrogen metabolism in fungi ([Fu and Marzluf, 1990](#_ENREF_2); [Giese et al, 2013](#_ENREF_3)) |
| FGSG_09565 ^2^ | probable siderophore regulation protein (GATA factor) | SREA | Homeostasis of iron in Aspergillus ([Schrettl et al, 2008](#_ENREF_7)) |
| FGSG_13123 ^6^ | related to finger protein AZF1 | AZF1 | Involved in diauxic shift; regulates genes involved in growth and carbon metabolism in the presence of glucose; involved in maintenance of cell wall integrity ([Newcomb et al, 2002](#_ENREF_5); [Slattery et al, 2006](#_ENREF_8); [Stein et al, 1998](#_ENREF_10)) |
|  |  |  |  |

Feng B, Haas H, Marzluf GA (2000). ASD4, a New GATA Factor of Neurospora crassa, Displays Sequence-Specific DNA Binding and Functions in Ascus and Ascospore Development†. *Biochemistry* **39:** 11065-11073.

Fu YH, Marzluf GA (1990). nit-2, the major positive-acting nitrogen regulatory gene of Neurospora crassa, encodes a sequence-specific DNA-binding protein. *Proc Natl Acad Sci U S A* **87:** 5331-5335.

Giese H, Sondergaard TE, Sorensen JL (2013). The AreA transcription factor in Fusarium graminearum regulates the use of some nonpreferred nitrogen sources and secondary metabolite production. *Fungal Biol* **117:** 814-821.

Maoz N, Gabay O, Waldman Ben-Asher H, Cohen HY (2014). The Yeast Forkhead HCM1 Controls Life Span Independent of Calorie Restriction. *The journals of gerontology Series A, Biological sciences and medical sciences*.

Newcomb LL, Hall DD, Heideman W (2002). AZF1 is a glucose-dependent positive regulator of CLN3 transcription in *Saccharomyces cerevisiae*. *Mol Cell Biol* **22:** 1607-1614.

Pramila T, Wu W, Miles S, Noble WS, Breeden LL (2006). The Forkhead transcription factor Hcm1 regulates chromosome segregation genes and fills the S-phase gap in the transcriptional circuitry of the cell cycle. *Genes Dev* **20:** 2266-2278.

Schrettl M, Kim HS, Eisendle M, Kragl C, Nierman WC, Heinekamp T *et al.* (2008). SreA-mediated iron regulation in Aspergillus fumigatus. *Molecular Microbiology* **70:** 27-43.

Slattery MG, Liko D, Heideman W (2006). The function and properties of the Azf1 transcriptional regulator change with growth conditions in Saccharomyces cerevisiae. *Eukaryot Cell* **5:** 313-320.

Son H, Seo Y-S, Min K, Park AR, Lee J, Jin J-M *et al.* (2011). A Phenome-Based Functional Analysis of Transcription Factors in the Cereal Head Blight Fungus, <italic>Fusarium graminearum</italic>. *PLoS Pathog* **7:** e1002310.

Stein T, Kricke J, Becher D, Lisowsky T (1998). Azf1p is a nuclear-localized zinc-finger protein that is preferentially expressed under non-fermentative growth conditions in Saccharomyces cerevisiae. *Curr Genet* **34:** 287-296.
