# Supplementary material for: Fungal Innate Immunity Induced by Bacterial Microbe-Associated Molecular Patterns (MAMPs)
Source: G3 (Bethesda). 2016 Mar 29;6(6):1585–95. doi: 10.1534/g3.116.027987 (PMC4889655; doi:10.1534/g3.116.027987)
Supplement: Supplemental Material [file supp_g3.116.027987_TableS16.doc]

Table S16: The table below (and illustrated as a figure) shows the Log 2 fold change of genes encoding LRR-containing adenylate cyclases (ATP pyrophosphate-lyase) or lyase domains in responses to MAMPs in relation water control in *Fusarium graminearum*. None of these proteins are predicted to be membrane proteins. Even though the responses are weak as could be expected for receptor type proteins, they could be part of the MAMPs recognition machinery as previously hypothesized .

|  | FLG1 | FLG2 | FLG4 | LOS1 | LOS2 | LOS4 | PGN1 | PGN2 | PGN4 | LRRs | Gene Description |
| --- | --- | --- | --- | --- | --- | --- | --- | --- | --- | --- | --- |
| FGSG_01522 | 1.070015 | 0.486171 | 0.123886 | -0.89486 | -0.26391 | -0.14975 | 0.252892 | -0.03961 | 0.266776 | 15 | probable regulatory subunit of protein phosphatase-1 |
| FGSG_01645 | -0.02802 | -0.1241 | 0.111442 | 0.265487 | 0.256524 | 0.719399 | -0.17435 | 0.009152 | -0.2342 | 17 | conserved hypothetical protein |
| FGSG_08285 | 0.309896 | -0.11772 | 0.022049 | 0.102685 | 0.166946 | 0.446249 | -0.03693 | 0.183362 | -0.03042 | 7 | related to receptor-like protein kinase 5 precursor |
| FGSG_01234 | 0.076683 | -0.08823 | -0.18902 | 0.432853 | 0.256593 | 0.244439 | 0.019419 | 0.173817 | 0.025833 | 28 | probable adenylate cyclase |
| FGSG_00627 | 0.257553 | -0.01082 | 0.003852 | 0.121787 | 0.058532 | 0.290193 | 0.054461 | 0.068436 | -0.06493 | 6 | related to adenylate cyclases |


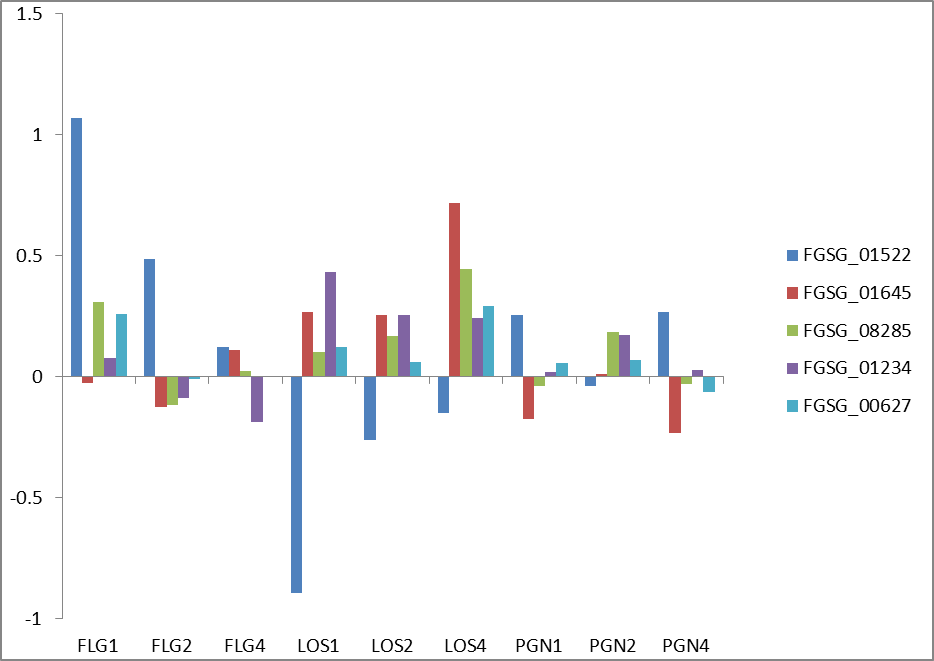


References:

Soanes DM, Talbot NJ (2010). Comparative genome analysis reveals an absence of leucine-rich repeat pattern-recognition receptor proteins in the kingdom Fungi*. PloS o*n**e** 5: e12725.
