# Supplementary material for: Fungal Innate Immunity Induced by Bacterial Microbe-Associated Molecular Patterns (MAMPs)
Source: G3 (Bethesda). 2016 Mar 29;6(6):1585–95. doi: 10.1534/g3.116.027987 (PMC4889655; doi:10.1534/g3.116.027987)
Supplement: Supplemental Material [file supp_g3.116.027987_FigureS1.pdf]

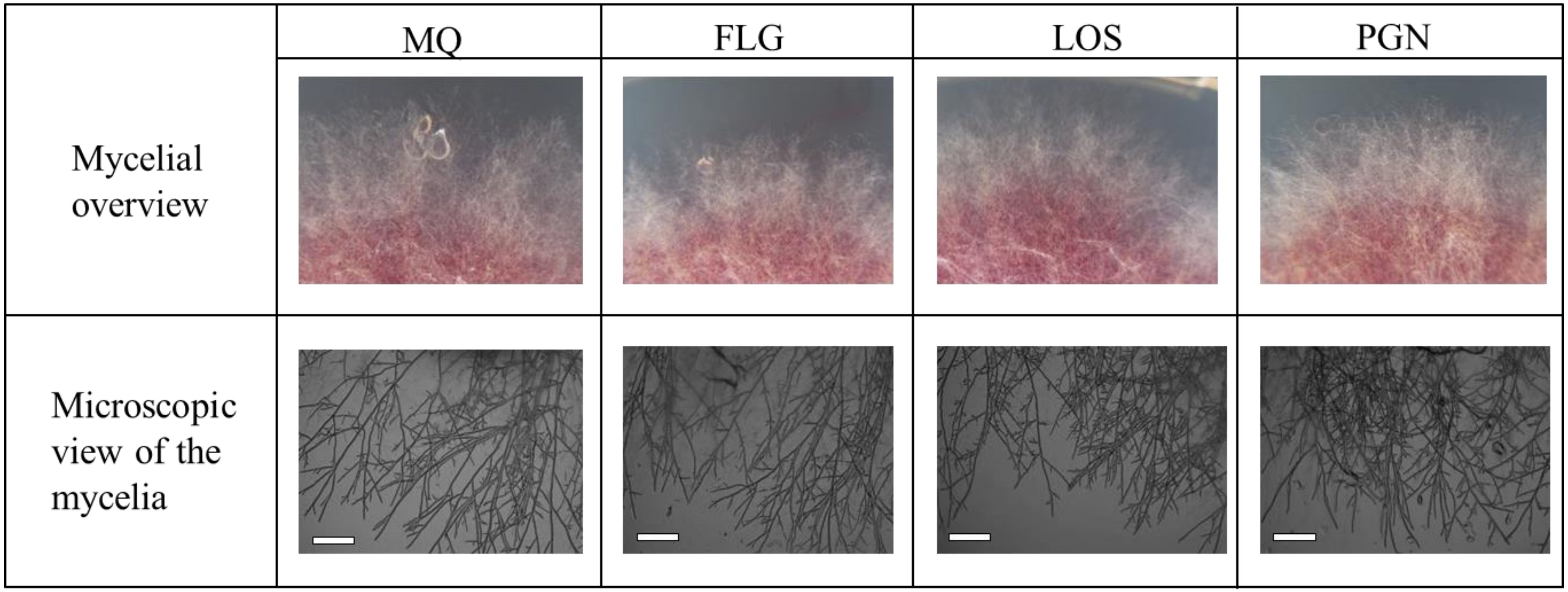

Figure S1: The effect of MAMPs on fungal cultures was tested.MAMPs (20ul) were deposited at the edge of fungal cultures on agar plates and allowed to grow onto the MAMPs inoculated area overnight. Exposure to MAMPs did not show any visible changes in colony or mycelium morphology. The scale bar on the microscopic pictures represents 0.4 mm.
