# Supplementary material for: Fungal Innate Immunity Induced by Bacterial Microbe-Associated Molecular Patterns (MAMPs)
Source: G3 (Bethesda). 2016 Mar 29;6(6):1585–95. doi: 10.1534/g3.116.027987 (PMC4889655; doi:10.1534/g3.116.027987)
Supplement: Supplemental Material [file supp_g3.116.027987_FigureS2.pdf]

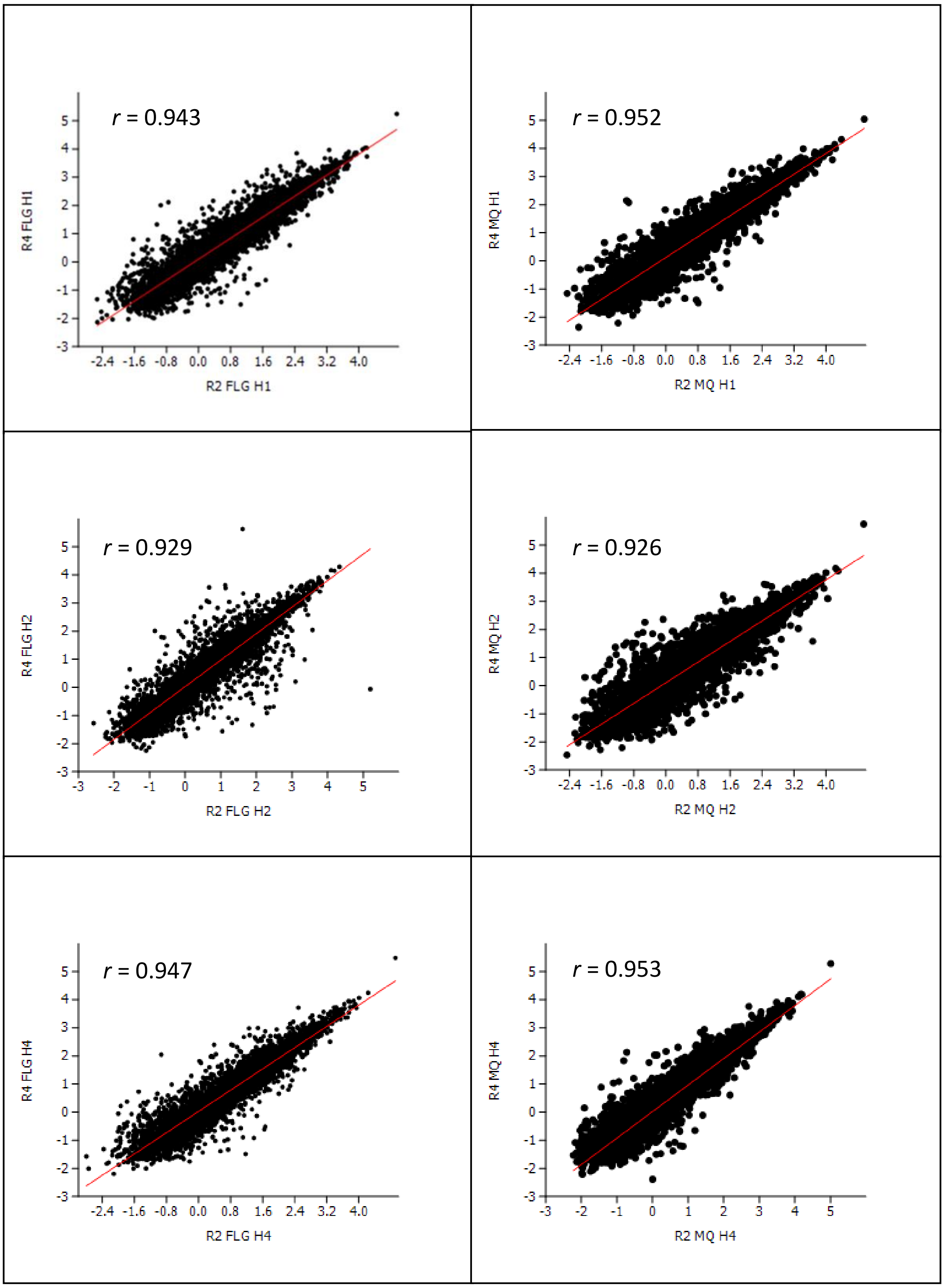

Figure S2: To validate the reproducibility of the fungal reactions, RNA-Seq expriments with flagellin (FLG) and water (MQ) were performed in two different laboratories with biological triplicates. The average expression values (FPKM) from each treatment was compared to each other for correlations. The calculated Pearson's correlation efficient *r* was above 0.92 for all the treatments as illustrated above, hence showing reproducibility. Slight variation in the *r* value is to be expected due to the biological variations between the experiments. The data with "R2" prefix is described in this paper.
