# Supplementary material for: Fungal Innate Immunity Induced by Bacterial Microbe-Associated Molecular Patterns (MAMPs)
Source: G3 (Bethesda). 2016 Mar 29;6(6):1585–95. doi: 10.1534/g3.116.027987 (PMC4889655; doi:10.1534/g3.116.027987)
Supplement: Supplemental Material [file supp_g3.116.027987_FigureS3.pdf]

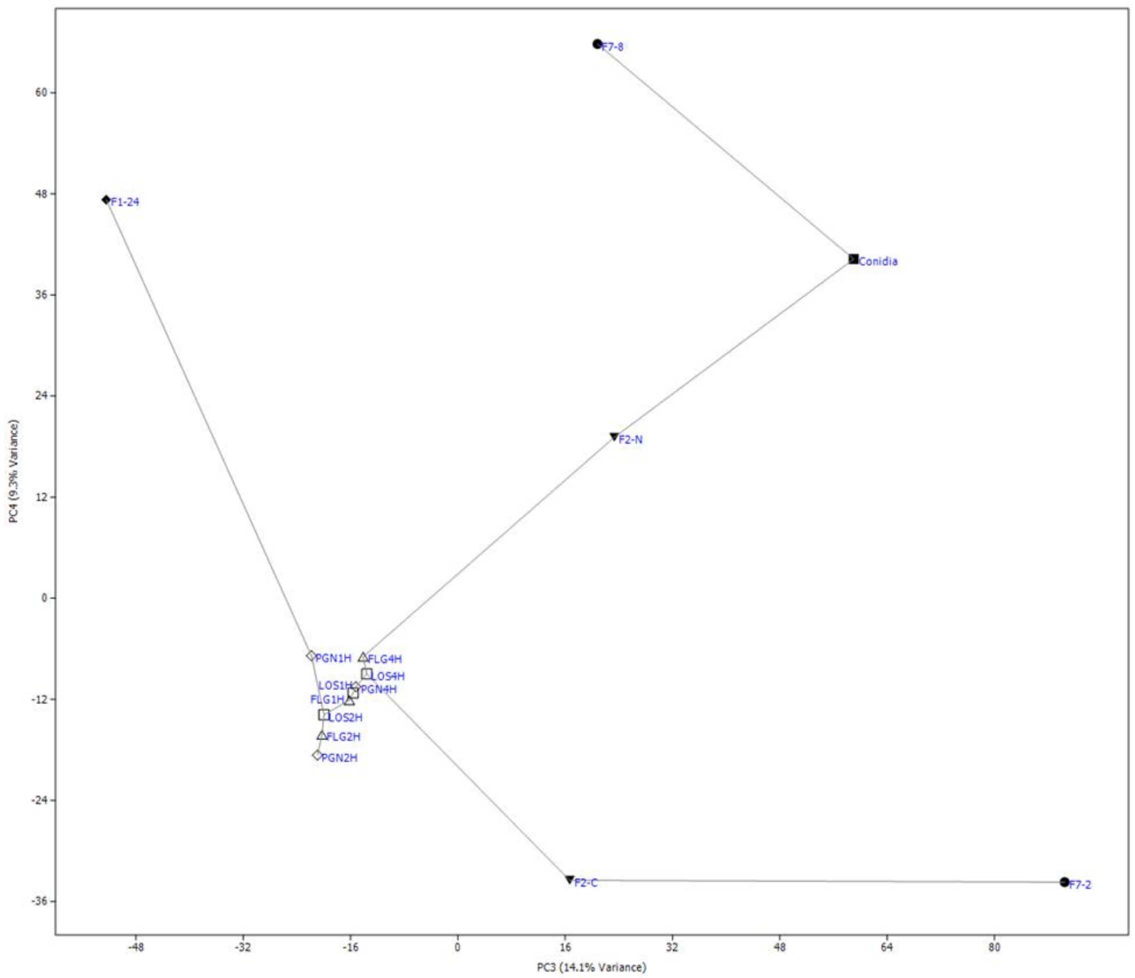

Figure S3:The figure above illustrates the relationships of the transcriptomics profiles from different experiments in relation to each other within the 3rd and 4th principle component capturing 14.1% variance and 9.3% variance respectively. The MAMPs samples still cluster together through PC3 and PC4 illustrating very close transcriptomics profiles.
