# Supplementary material for: Fungal Innate Immunity Induced by Bacterial Microbe-Associated Molecular Patterns (MAMPs)
Source: G3 (Bethesda). 2016 Mar 29;6(6):1585–95. doi: 10.1534/g3.116.027987 (PMC4889655; doi:10.1534/g3.116.027987)
Supplement: Supplemental Material [file supp_g3.116.027987_FileS1.pdf]

Supplementary materials and methods

This document provides additional information related to the material and methods section of the manuscript.

Assessing effect of MAMPs on fungal culture

Fungal cultures on DFM agar plates were grown in the dark at 21°C. When the culture was of reasonable size, a drop of 20µl MAMPs or water was inoculated 1cm from the edge of the culture and allowed to grow into the treated area overnight. The physiology of the fungal hyphae was observed the following day. Pictures of the hyphae growing on the inoculated area were taken using a cannon powershot ELPH330HS camera and an inverted microscope, Nikon eclipse TE300 at 4x magnification was used to observe if the hyphae suffered from any changes when in contact with MAMPs.

Fungal culture in microtitre plate

In each well of a microtitre plate, 1000 spores and 50x diluted DFM was incubated overnight at 21°C in the dark. The media was replaced with either 100µl of FLG or 100µl LOS or 100µl of a combination of FLG and LOS at testing concentrations. Each test was performed in at least 3 biological replicates. PGN was not tested due to lack of availability. Each well was supplemented with 20 ng DiOC7(3), 3,3′ Diheptyloxacarbocyanine iodide (D0929 SIGMA). Fluorometric monitoring was performed as explained below

Fluorometric detection

Fluorometric monitoring was performed in a 4x4 matrix (1400 gain) per well using a FLUOstar OPTIMA plate reader (BMG Labtech, Ortenberg, Germany) with emission filters at 485nm and excitation filter at 520nm. Measurements were taken every 30 minutes for the duration of the experiment. The net effect of the MAMP(s) was calculated by subtracting the control. The average of the replicates and standard error values are reported

Mycelial production and MAMPs treatment

Fungal mycelia were cultured in 250 ml Erlenmeyer baffled flask (Bellco, NJ, USA) with 50 ml of DFM that was inoculated with 10 fungal plugs (approximately 8 mm x 8 mm). The flasks were shaken at 150 rpm for 3 days in the dark at 21°C. The fungal biomass was filtered through Miracloth and transferred into DFM without a nitrogen source for an overnight nitrogen depletion to recreate the nitrogen limiting environment of the soil. Three milliliters of the

resulting culture was filtered through Miracloth, rinsed with sterile MilliQ water prior to the desired MAMP or water.

The treatments were performed in biological triplicates in 13 ml Sarstedt tubes (Germany) with shaking in the dark at 21°C for 1, 2 and 4 hours. Post treatment, the cultures were filtered through Miracloth; snap frozen in liquid nitrogen, freeze dried and ground prior to RNA extraction.

Bioinformatics analysis

The sequencing results from the UMGC were stored on the servers of the Minnesota Supercomputing Institute (MSI) and they were analyzed using the Tuxedo analysis suit pipeline (Trapnell et al, 2012) through the Galaxy web interface (Blankenberg et al, 2010b; Giardine et al, 2005; Goecks et al, 2010) which was also hosted on the MSI servers. Briefly the raw data was checked for quality using FASTQC (Andrews, 2010), groomed using FASTQ Groomer (v1.0.4) (Blankenberg et al, 2010a), mapped with Tophat (v1.5) (Trapnell et al, 2009) and differential expression analysis (including the adjusted *t*-test for significance) was performed using Cuffdiff (Trapnell et al, 2010). The level of expression is expressed as Fragments Per Kilobase Of Exon Per Million Fragments Mapped (FPKM). Genes were considered as differentially expressed if they had more than 2 fold expression differences and had an adjusted confidence level that was greater than 95%.

The genome sequence for *F. graminearum* and other relevant files were obtained from the BROAD website (http://www.broadinstitute.org/annotation/genome/fusarium_group/MultiHome.html). Intron size values to fine-tune the Tuxedo softwares were obtained from the analysis of the genome sequence (Cuomo et al, 2007). Additional genome related information such as 1000bp sequences upstream of genes start site were obtained from the BROAD website. Pfam domains and FunCat that are associated with genes were obtained from the MIPS *Fusarium graminearum* Genome Database (FGDB) (http://mips.helmholtz-muenchen.de/genre/proj/FGDB/). Data was processed using Ms Excel and Ms Access.

Promoter sequences from genes that were significantly regulated were extracted using FASTA sequence extractor from FaBox(Villesen, 2007) and searched for transcription factor motifs using Multiple EM for Motif Elicitation (MEME) analysis suite v 4.9.1 (Bailey et al, 2009). The potential transcription factor binding to the motif was searched using the TOMTOM comparison tool (Gupta et al, 2007) against all yeast promoter databases. The ortholog of the yeast transcription factor was searched against the fungal genome using a protein sequence similarity search (Altschul et al, 1990).

Correlation analysis

The experiments with FLG and water were repeated in biological replicates in two different laboratories. To compare the reproducibility between the experiments, the average expression of each gene (FPKM) from each experiments were plotted using the PAST analysis software (Hammer et al, 2001) and fitted using a bivariate linear model. Prior to the analysis, the datasets were trimmed by removing genes with expression values of zero and the expression level were transformed to log 10. The Pearson's correlation value indicating the correlation between the two experiments is reported.

Principal Component Analysis (PCA) with a correlation matrix was performed using the PAST analysis software. Publically available expression datasets were accessed from www.plexdb.org. Experiments FG1(fungal infection 24hr post inoculation in barley spikes against mock infection) (Guldener et al, 2006), FG2 (Fungi grown in medium with no carbon source or no nitrogen source as compared to complete medium growth) (Guldener et al, 2006), FG7 (germinated conidia after 2 hours compared to non-germinated conidia, and 8hr germinated conidia compared to 2hr germinated conidia) (Seong et al, 2008) and Conidia (non-germinated conidia compared to mycelium) (Zhao et al, 2014) were additionally used for comparisons against the MAMPs datasets since they were closest in physiology and time range to the MAMPs treatments. The fold change compared to controls (Log 2 values) was used for the PCA to ease the comparison between the two different technological platforms.

References

Altschul SF, Gish W, Miller W, Myers EW, Lipman DJ (1990). Basic local alignment search tool. *J Mol Biol* **215:** 403-410.

Andrews S (2010). FastQC: A quality control tool for high throughput sequence data.http://www.bioinformatics.babraham.ac.uk/projects/fastqc/

Bailey TL, Boden M, Buske FA, Frith M, Grant CE, Clementi L *et al.* (2009). MEME SUITE: tools for motif discovery and searching. *Nucleic Acids Res* **37:** W202-208.

Blankenberg D, Gordon A, Von Kuster G, Coraor N, Taylor J, Nekrutenko A (2010a). Manipulation of FASTQ data with Galaxy. *Bioinformatics* **26:** 1783-1785.

Blankenberg D, Von Kuster G, Coraor N, Ananda G, Lazarus R, Mangan M *et al.* (2010b). Galaxy: a web-based genome analysis tool for experimentalists. *Curr Protoc Mol Biol* **Chapter 19:** Unit 19 10 11-21.

Cuomo CA, Guldener U, Xu JR, Trail F, Turgeon BG, Di Pietro A *et al.* (2007). The *Fusarium graminearum* genome reveals a link between localized polymorphism and pathogen specialization. *Science* **317:** 1400-1402.

Giardine B, Riemer C, Hardison RC, Burhans R, Elnitski L, Shah P *et al.* (2005). Galaxy: a platform for interactive large-scale genome analysis. *Genome Res* **15:** 1451-1455.

Goecks J, Nekrutenko A, Taylor J, Galaxy T (2010). Galaxy: a comprehensive approach for supporting accessible, reproducible, and transparent computational research in the life sciences. *Genome Biol* **11:** R86.

Guldener U, Seong KY, Boddu J, Cho S, Trail F, Xu JR *et al.* (2006). Development of a *Fusarium graminearum* Affymetrix GeneChip for profiling fungal gene expression in vitro and in planta. *Fungal Genet Biol* **43:** 316-325.

Gupta S, Stamatoyannopoulos J, Bailey T, Noble W (2007). Quantifying similarity between motifs. *Genome Biol* **8:** R24.

Hammer Ø, Harper DAT, Ryan PD (2001). PAST: Paleontological statistics software package for education and data analysis. *Palaeontologia Electronica* **4:** 9.

Seong KY, Zhao X, Xu JR, Guldener U, Kistler HC (2008). Conidial germination in the filamentous fungus *Fusarium graminearum*. *Fungal Genet Biol* **45:** 389-399.

Trapnell C, Pachter L, Salzberg SL (2009). TopHat: discovering splice junctions with RNA-Seq. *Bioinformatics* **25:** 1105-1111.

Trapnell C, Williams BA, Pertea G, Mortazavi A, Kwan G, van Baren MJ *et al.* (2010). Transcript assembly and quantification by RNA-Seq reveals unannotated transcripts and isoform switching during cell differentiation. *Nat Biotechnol* **28:** 511-515.

Trapnell C, Roberts A, Goff L, Pertea G, Kim D, Kelley DR *et al.* (2012). Differential gene and transcript expression analysis of RNA-seq experiments with TopHat and Cufflinks. *Nat Protoc* **7:** 562-578.

Villesen P (2007). FaBox: an online toolbox for fasta sequences. *Molecular Ecology Notes* **7:** 965-968.

Zhao C, Waalwijk C, de Wit PJ, Tang D, van der Lee T (2014). Relocation of genes generates non-conserved chromosomal segments in *Fusarium graminearum* that show distinct and co-regulated gene expression patterns. *BMC Genomics* **15:** 191.
